# Effects of a Passive Online Software Application on Heart Rate Variability and Autonomic Nervous System Balance

**DOI:** 10.1089/acm.2016.0198

**Published:** 2017-01-01

**Authors:** Beverly Rubik

**Affiliations:** ^1^ Institute for Frontier Science, Oakland, CA.; ^2^ Energy Medicine University, Sausalito, CA.; ^3^ College of Integrative Medicine and Health Sciences, Saybrook University, Oakland, CA.

**Keywords:** heart rate variability, biofield, energy, autonomic nervous system, stress

## Abstract

***Objective:*** This study investigated whether short-term exposure to a passive online software application of purported subtle energy technology would affect heart rate variability (HRV) and associated autonomic nervous system measures.

***Methods:*** This was a randomized, double-blinded, sham-controlled clinical trial (RCT). The study took place in a nonprofit laboratory in Emeryville, California. Twenty healthy, nonsmoking subjects (16 females), aged 40–75 years, participated. Quantum Code Technology^™^ (QCT), a purported subtle energy technology, was delivered through a passive software application (Heart+ App) on a smartphone placed <1 m from subjects who were seated and reading a catalog. HRV was measured for 5 min in triplicate for each condition via finger plethysmography using a Food and Drug Administration medically approved HRV measurement device. Measurements were made at baseline and 35 min following exposure to the software applications. The following parameters were calculated and analyzed: heart rate, total power, standard deviation node-to-node, root mean square sequential difference, low frequency to high frequency ratio (LF/HF), low frequency (LF), and high frequency (HF).

***Results:*** Paired samples *t*-tests showed that for the Heart+ App, mean LF/HF decreased (*p* = 9.5 × 10^–4^), while mean LF decreased in a trend (*p* = 0.06), indicating reduced sympathetic dominance. Root mean square sequential difference increased for the Heart+ App, showing a possible trend (*p* = 0.09). Post–pre differences in LF/HF for sham compared with the Heart+ App were also significant (*p* < 0.008) by independent *t*-test, indicating clinical relevance.

***Conclusions:*** Significant beneficial changes in mean LF/HF, along with possible trends in mean LF and root mean square sequential difference, were observed in subjects following 35 min exposure to the Heart+ App that was working in the background on an active smartphone untouched by the subjects. This may be the first RCT to show that specific frequencies of a purported non-Hertzian type of subtle energy conveyed by software applications broadcast from personal electronic devices can be bioactive and beneficially impact autonomic nervous system balance.

## Introduction

Unmitigated chronic stress profoundly affects health. It is one of the most important factors underlying all chronic diseases, morbidity, mortality, immune-suppression, mood disorders, and work absenteeism.^1–3^ Approximately 80% of the health problems in technologically advanced societies are considered to be stress related.^4^ Although there are established techniques to manage stress effectively, each has its limitations. For example, relaxation-based approaches such as meditation and focused breathing have been shown to be beneficial, but these require training and time investments that may prohibit high compliance rates.^5^ Physical exercise is another effective approach, but again, due to the time commitment, compliance is often low.^6^ Thus, new strategies to protect against the ravages of chronic stress are sorely needed.

Sympathetic Resonance Technology^™^ (SRT), a purported subtle energy technology, is one such passive modality that has been shown to impact the stress response without any effort on the part of the user. SRT is used in consumer healthcare products that are placed on or nearby the subject, such as the QLink^®^ pendant (QLink^®^ SRT Products, Bellevue, WA). Previous studies on a variety of living systems exposed to known stressors have shown that the stress response was measurably decreased by SRT. A variety of protective effects from SRT to various environmental stressors have been shown, including protection of fibroblast cultures, reduced oxidative stress in the blood of humans, decreased acupuncture meridian stress, reduced state anxiety scores in school children,^7^ and electroencephalograph stabilization in humans.^8^ The present study tests new and improved generation technology derived from SRT produced by the same inventors, Robert O. Williams and Patti Leach, who digitized it using a proprietary method. This new digital form, Quantum Code Technology^™^ (QCT), was enclosed in a software application by ONE08, Inc., the Heart+ App. Transmission over the Internet of the Heart+ App between the United States and Australia showed no loss of signal integrity (Williams RO, Kelly MPJ, Fisher M, unpublished data, 2015). Both SRT and the Heart+ App involve proprietary frequencies protected by trade secret law. SRT and the Heart+ App involve no measurable electric, magnetic, or electromagnetic fields.

Although there is no presently accepted scientific basis for subtle energy interactions, the rudiments of a scientific foundation involving an interaction with the biofield have been previously proposed.^7,9–12^ The term “biofield” (or “bio field”), a medical subject heading at the U.S. National Library of Medicine since 1995, describes the biologic field of the organism proposed to be dynamic and integrative (integrating numerous sources of endogenous and exogenous fields via the superposition principle). The biofield is comprised of electromagnetic, electric, magnetic, and acoustic fields. It is hypothesized to be a super-regulator of the physiology and biochemistry of the organism, capable of sustaining homeodynamics and thereby essential to maintaining health and wellness.^9–13^ When energy-field stimuli interact with the biofield, they may produce resonance, entrainment, or otherwise perturb it. An altered biofield is expected to lead to a shift in the homeodynamics and physiology of the organism.^9,10^ “Subtle energy” refers to unconventional energy fields that are fundamentally undetectable using conventional physical measurement devices. One scientific basis for subtle energy arises in the potentials in Maxwell's original electromagnetic field equations.^14^ According to this theory, an electromagnetic field was described by five components: velocity of propagation, electric field, magnetic field, electric scalar potential, and magnetic scalar potential. In 1893, a simplification of the Maxwell equations was published by Oliver Heaviside in his classical textbook.^15^ Heaviside preserved the Hertzian components (transverse waves) of electromagnetism, but eliminated the scalar and vector potentials because he considered them insignificant for applications. The Heaviside formulation has been promulgated ever since in electromagnetism textbooks. However, these potentials were never eliminated from quantum theory. The Aharonov–Bohm effect shows a real quantum effect from an electromagnetic potential.^16^ An electrically charged particle is affected by a potential in a region where both the magnetic and electric fields are zero.^17^ Subtle energies based on these potentials are called “scalar fields,” “longitudinal fields,” or “non-Hertzian fields.” SRT and QCT purportedly involve non-Hertzian fields.

Information in living systems is key, whether it is biomolecular in form or conveyed through an electromagnetic field parameter as “electromagnetic bioinformation.”^18^ Extremely weak, nonionizing, conventional electromagnetic fields (Hertzian) whose energy content is below the physical thermal noise limit (of random molecular motion) may have biological effects by virtue of the information these waves transmit, encoded in field parameters such as frequency, modulation, phase, degree of coherence, and so on,^19–22^ and the evidence that organisms are dynamic nonlinear nonequilibrium systems exquisitely sensitive to extremely weak electromagnetic signals. These signals may impact the biofield or specific processes in the organism, for example, by altering the dynamics of receptor–ligand interactions on the cell membrane. There is speculation that non-Hertzian fields may convey information of specific field parameters such as frequency that impacts organisms in a similar manner.^12,23,24^ Thus, the Heart+ App is thought to transmit the information of key frequencies to achieve resonance with the biofield.^7^ The QCT frequencies in the Heart+ App were found to affect the human biofield measurably in acupuncture meridian conductivities at distances of up to 10 m (Taylor DB, unpublished data, 2014–2015). Such effects may increase the dynamic stability of the biofield, which is constantly challenged by numerous external influences from the environment as well as from internal dynamic fluctuations. The biofield, thus strengthened with respect to key frequencies, may then produce beneficial effects on the whole organism, as the biofield is hypothesized to be fundamentally involved in biological regulation.^7,9^

Subtle energy effects on biological systems have been measured by others. Ho et al. showed that the magnetic potential can alter pattern formation in Drosophila embryos.^25^ Bioinformation of a plant growth hormone transmitted by non-Hertzian electromagnetic signals was shown to increase the growth of plants.^26^

Over the past decade, numerous consumer healthcare products have emerged that involve frequency information and/or subtle energy fields that claim to have beneficial health effects.^13^ Yet, hardly any of these products have been clinically tested by independent laboratories. This report documents results of the first clinical trial on the Heart+ App, a digitized proprietary informational signal of key frequencies broadcast over the Internet. This study investigated the short-term effects of the Heart+ App on heart rate variability (HRV) of human subjects under rigorously controlled experimental conditions.

HRV is more than just an indicator of heart health; it is regarded as an important biomarker of stress, autonomic nervous system (ANS) status, and a vital sign of overall health.^27^ A power spectral analysis of the R–R interval (beat-to-beat) variations of the heart rate (HR) is widely used to quantify cardiac autonomic regulation.^28–30^ This analytical method partitions the total variance of a continuous series of beats into its frequency components, identifying three main peaks: very low frequency (VLF), <0.04 Hz; low frequency (LF), 0.04–0.15 Hz; and high frequency (HF), 0.15–0.4 Hz. Increases in LF/HF are generally considered to reflect a shift to “sympathetic dominance,” whereas decreases in LF/HF generally correspond to a reduction in sympathetic dominance and/or an increase in parasympathetic function. However, there is some debate over this interpretation,^31^ which will be further developed in the Discussion section. Typical autonomic imbalance is characterized by a hyperactive sympathetic system and a hypoactive parasympathetic system due to conditioning of the stress response, and is associated with various pathological conditions.^29^ Measurements of HRV are becoming more widely known and used in research and clinical practice.

Preliminary data on SRT influencing HRV parameters are available from two unpublished pilot studies. In the first study, a single subject was tested with an AC electric clock treated with SRT placed 1 m away, using HRV and electrocardiogram testing. Results show that the presence of SRT correlated with increased HRV and improved balance in the power spectrum between the LF and HF bands (Tiller W, unpublished data. Experimental results for Clarus ClearWave Clock conducted May 4, 1993, Boulder Creek, CA). In the second study, 18 female subjects (*M*_age_ = 26.5 years) wearing a QLink pendant with SRT compared with placebo (sham device) were tested without an imposed stressor. There was a statistically significant increase in one measure of HRV (standard deviation node-to-node [SDNN]) after 1 h exposure to SRT, and an increasing tendency in another measure of HRV (root mean square sequential difference [RMSSD]; Choi C, unpublished data, 2005). These findings are consistent with a reduced stress response and/or improved ANS balance between the sympathetic and parasympathetic functions.

Preliminary testing of the Heart+ App on >100 subjects was previously demonstrated by Dr. Daniel B. Taylor, OMD, ND, of Melbourne, Australia. He found that the acupuncture meridian stress of test subjects measured with the BioMeridian MSAS (International Health Technologies, Salt Lake City, UT) was rapidly reduced upon exposure to the Heart+ App. The electrical conductivity of the major meridians that had been abnormally high or low shifted to normal values in the presence of the Heart+ App (Taylor DB, unpublished data, 2015). This result was independently replicated on several subjects by this author (data not shown).

### Study design

This was a randomized, double-blind, sham-controlled pilot study in which each subject received both sham and test conditions (i.e., a within-group design). Subjects were randomly assigned to receive either the Heart+ App or the sham app first. The order of intervention was therefore counterbalanced to control for any ordering effects. The study was designed to look for any changes in resting HRV, measured pre- and post-intervention, due to the effects of the Heart+ App or sham app. Because the study was exploratory, the effect size was initially unknown, and thus optimal sample size could not be predetermined.

## Materials and Methods

### Intervention and double-blinding

The Heart+ App and sham app appeared as identical software applications on identical smartphones (Apple, Inc., iPhone 6) encoded by phone case color. Subjects and researchers were blinded as to the code until the study was completed and data analyzed. The same network provider (T-Mobile 4G LTE) was used for each smartphone, thus controlling for any differences in signal strength due to the distance from the cell phone tower. The phones were switched on, ready to receive a call or text, but remained idle. They were broadcasting only through the cell phone network carrier frequencies. The sham app and Heart+ App were activated by a touch screen on the two different smartphones, respectively, and were completely passive, such that once the touch-screen software switch was turned on, the apps ran automatically without any further input. The smartphones were operated by the researcher to deliver the test and sham interventions, which were each 35 min in duration. No subject handled either smartphone, which was placed on the desk directly in front of the subjects, <1 m from them, where subjects could see the phones. The apps were not seen by the subjects, however, because the touch-screens were turned off. Upon software activation, the frequency information of non-Hertzian QCT signals was broadcast by the Heart+ App and received by the subject, whereas the sham app looked identical but did not transmit any signal. Both the Heart+ App and the smartphone were turned off at the end of each intervention period and stored in a Faraday cage.

### Control of ambient electromotive forces

Ambient electromotive forces of the experimental room were reduced by removing nonessential laboratory and office equipment. The Mersmann ELF Fieldmeter (Bio-Physik Mersmann, Wassenach, Germany) was used to measure ambient electric and magnetic fields using different antennae and coils. When placed at the test site, it indicated that DC electric = 0 V/m and magnetic fields <10 nT for up to 300 Hz. VLF fields = 0 mV, as measured with the ME3951A (Gigahertz Solutions, Fürth, Germany), and a radio frequency meter, Cornet ED-65 (Cornet Microsystem, Inc., Santa Clara, California), measured <−35 dBm. The same location in the laboratory was used for all experimental runs. No WiFi-enabled Internet modem was present. All portable electronic devices not in use during the study, including those of the subjects, were turned off and stored in a Faraday cage to shield against any microwave bursts associated with wireless communications. It was verified that these devices in the Faraday cage could not be remotely activated.

### Subjects

Volunteers (*N* = 20) were healthy adults, without chronic diseases or conditions, including psychiatric, and not taking medications except for birth control hormones and/or hormone replacement therapy. Subject inclusion criteria were 40–75 years of age, nonsmoking, not pregnant or trying to get pregnant, and having a body mass index <30 (i.e., non-obese). Exclusion criteria were recreational drug use, heart pacemakers or other powered implants, electrohypersensitivity to cell phones and/or other consumer electronic devices, and long-term (>2 years) regular practice of meditation, yoga, and/or qigong. These criteria were established to obtain a sample of diverse, healthy adults, middle aged to elderly, characteristic of the general population, where chronic stress is often a health challenge. Those with long-term mind–body practices were excluded because this subpopulation shows exceptionally good autonomic regulation and would be expected to be less responsive to external influences compared with the general population.

Recruitment was done via advertising on Craigslist.org, announcements on local college e-mailing lists, LinkedIn.com, Facebook.com, and by word-of-mouth in the San Francisco Bay Area. Prospective subjects completed a participant screen that predetermined their eligibility for the study.

The sample demographics (*N* = 20) were a mean age of 57.2 years (range 40–75 years), comprised of 6 males and 14 females, and consisted of 15 Caucasians, 3 Asians, and 2 African Americans. The study was pre-approved by an Institutional Review Board. All participants gave written informed consent.

### Procedures

The experimental sessions were conducted from August to October 2015. Each subject was randomly assigned to receive either the Heart+ App or the sham app first, and to receive the other intervention on a different day, yielding two sessions per subject on different days at the same time. Each subject's sessions were conducted at the same time of day to control for any differences due to circadian rhythms. Subject personal electronic devices were turned off and stored in a Faraday cage along with any subject jewelry. Each session was conducted in the same location in the laboratory.

Subjects came by individual appointment and were informed about the purpose and protocol for the study both on the consent form and by means of the researcher using a standard verbal script. Upon arrival, each was seated and given neutral reading material, a catalog of outdoor goods, to read for 15 min. Subjects were in the same room with the researcher so that their reading activity during each run was monitored. This reading material was used throughout the study for all experimental runs. Then HRV was measured using a Medicore SA-3000P (Medicore Co. Ltd., Seoul, Korea), an FDA medically approved device, via finger plethysmography for 5 min, in triplicate, constituting the baseline measurements. The subject was then exposed to a smartphone in active mode running either the Heart+ App or sham app, placed <1 m distance from the body for 35 min. The initial exposure condition was randomly assigned by use of a random number table. The number of subjects who received the Heart+ App first and the sham app first were identical to control for any ordering effect. Each subject read the same catalog during the 35 min exposure condition. Then HRV was measured in triplicate again for 5 min periods, constituting the test measurements. Thus, subject participation consisted of sitting, reading, and having repeated 5 min pulse measurements taken from the same finger. No subject touched either smartphone. At the end of each session, each subject was compensated for her/his time and departed.

### Measures

Table 1 shows the HRV parameters that were analyzed. Time- and frequency-domain parameters of HRV were calculated from the pulse tachogram. The time domain parameters are HR, SDNN, and RMSSD. From the power spectrum analysis, the total power (TP), LF band, and the HF band as well as the LF/HF ratio are calculated. The Medicore HRV software computed these parameters for every 5 min measurement.

**Table 1.** HRV Parameters, Units, and Basic Relationship to ANS Function

*HRV parameter*	*Acronym, units*	*Association*
Heart rate	HR, bpm	Responsive to ANS function
Standard deviation node-to-node	SDNN, ms	HRV, time domain measurement
Root mean square sequential difference in R–R intervals	RMSSD, ms	Short-term value of HRV
Total power	TP, ms^2^	Total spectral power; total activity of neuro-humoral influences on heart rate
Low frequency to high frequency power ratio	LF/HF	Healthy range 0.5–2.5 indicates ANS balance and resilience to stress
Low frequency power, 0.04–0.15 Hz	LF, nu	Indicator of sympathetic function plus some parasympathetic function
High frequency power, 0.15–0.4 Hz	HF, nu	Indicator of parasympathetic function

HRV, heart rate variability; ANS, autonomic nervous system; bpm, beats per minute; ms, milliseconds; Hz, Hertz; nu, normalized units; HF, high frequency; LF, low frequency; LF/HF, low frequency to high frequency ratio; RMSSD, root mean square sequential difference; SDNN, standard deviation node-to-node; TP, total power.

## Results

A total of 240 HRV measurements were made. Of these, 60 were pre–Heart+ App, 60 post–Heart+ App, 60 pre-sham app, and 60 post-sham app. The data were analyzed using two-tailed paired samples *t*-tests. The independent variable was the treatment condition (Heart+ App and sham app), and the dependent variables were the HRV parameters shown in Table 1.

Table 2 shows the mean values and standard error of the mean for all subjects in both conditions, pre–post sham app and pre–post Heart+ App, as well as the calculated *p*-values from *t*-tests. Mean HR dropped approximately 2 bpm following the sham app (*p* = 6.3 × 10^–5^) and Heart+ App interventions (*p* = 0.0013), suggesting that a mild relaxation response occurred for both conditions. Mean LF/HF decreased significantly for the Heart+ App from 3.91, a value indicating autonomic imbalance, to 2.73 (*p* = 9.5 × 10^–4^), a decrease of 30.2%, while for the sham app, LF/HF increased 3.6%, from 3.05 to 3.16, which is insignificant (*p* = 0.74). Moreover, for the Heart+ App, mean LF decreased from 70.45 to 65.33 (*p* = 0.060), a decrease of 7.3%, indicating a strong trend. A small increase in LF was observed for the sham app that was insignificant (*p* = 0.29). The significant drop in mean LF/HF along with the trend in decreased mean LF suggests that the Heart+ App improved autonomic balance by reducing sympathetic dominance. Moreover, the post–Heart+ App LF/HF ratio of 2.73 is closer to the normal range of 0.5–2.5 that is considered optimal for ANS balance. However, the Heart+ App condition did not significantly change the value of HF (*p* = 0.78), such that no increase in parasympathetic function was observed. Mean RMSSD showed a possible trend for the Heart+ App, increasing from 26.58 to 28.48 (*p* = 0.090), but an insignificant change for the sham app (*p* = 0.24). No significant changes in any other variables were found for the sham app.

**Table 2.** Mean Values, Standard Error of the Mean, and *p*-Values of all HRV Parameters for the Sham App and Heart+ App Conditions

	*Sham app*					*Heart+ App*				
	*Pre sham*		*Post sham*			*Pre app*		*Post app*		
*HRV parameter*	*Mean*	*Std error*	*Mean*	*Std error*	p*-Value*	*Mean*	*Std error*	*Mean*	*Std error*	p*-Value*
HR	72.46	1.31	70.42	1.39	6.3 × 10^–5^^*^	73.57	1.46	71.19	1.46	0.0013^*^
SDNN	40.35	3.21	40.62	2.84	0.89	40.69	3.92	43.29	4.21	0.30
RMSSD	26.49	1.97	27.13	2.15	0.48	26.58	2.96	28.48	2.78	0.090
TP	1423.54	231.57	1832.59	349.83	0.079	1399.43	214.37	1462.51	232.37	0.71
LF/HF	3.05	0.38	3.16	0.36	0.74	3.91	0.44	2.73	0.30	0.00095^*^
LF	61.93	2.51	67.12	1.89	0.29	70.45	2.02	65.33	1.96	0.060
HF	38.07	2.51	32.89	1.89	0.11	32.29	2.53	33.32	1.93	0.78

^*^ *p*-Values obtained from paired samples *t*-tests are statistically significant at *p* < 0.05.

HR, heart rate.

The post–pre differences in LF/HF for the sham and Heart+ App were statistically compared. A *t*-test of two independent sample means was carried out on the post minus pre values of LF/HF for the sham app and the Heart+ App, using alpha = 0.05 and hypothesized mean difference = 0 (null hypothesis). This was calculated in two different ways, assuming equal variances and then unequal variances, although the results from these two methods were not significantly different. The results for the unequal variances case show that for two-tailed tests, p < 0.008 (Table 4). Therefore, the post–pre differences in LF/HF for the sham app compared with the Heart+ App are statistically significant by this test. The observed changes in LF/HF for the Heart+ App compared with the sham app are therefore nontrivial and appear to have clinical relevance.

### Statistical analysis

Mean values, standard deviations, and standard error of the mean were calculated for the pre–post conditions of the sham app and the Heart+ App. Two-tailed paired samples *t*-tests were run for all dependent variables to obtain the *p*-values shown in Table 2. Effect sizes were calculated using formulas for Cohen's *d* and Pearson's *r* and are listed in Table 3 for the significant outcomes and possible trends. The effect sizes are all small. Cohen's *d* = 0.195 and 0.21, and Pearson's *r* = 0.097 and 0.104, for the slight decrease in HR from the sham app and the Heart+ App, respectively. The effect sizes of the Heart+ App on RMSSD showed Cohen's *d* = 0.085 and Pearson's *r* = 0.043. The largest effect sizes were for the Heart+ App condition on LF/HF, with Cohen's *d* = 0.407 and Pearson's *r* = 0.2, and on LF, with Cohen's *d* = 0.332 and Pearson's *r* = 0.164. Table 4 shows the statistical results of the independent *t*-test comparing the means of two independent samples, and the post–pre differences in LF/HF for sham and Heart+ App, using alpha = 0.05 and assuming the null hypothesis. The results show *p* < 0.008 for the two-tailed test, indicating statistical significance.

**Table 3.** Calculated Effect Sizes, Cohen's *d*, and Pearson's *r* for Observed Significant Differences and Trends for the Sham App and Heart+ App Conditions

	*Sham app, pre–post*		*Heart+ App, pre–post*	
*HRV parameter*	*Cohen's* d	*Pearson's* r	*Cohen's* d	*Pearson's* r
HR	0.195	0.097	0.21	0.104
RMSSD			0.085	0.043
TP	0.178	0.089		
LF/HF			0.407	0.2
LF			0.332	0.164

**Table 4.** Comparison of the Means of the LF/HF Post–Pre Difference from the Sham App and the Heart+ App

	*Sham app LF/HF difference*	*Heart+ App LF/HF difference*
Mean	0.11105	−1.18119
Variance	6.711250625	6.913897339
Observations	60	60
Hypothesized mean difference		0
*df*		118
*t* Stat		−2.711740901
*p* (*T* ≤ *t*) one-tailed		0.00384669
*t*, critical one-tailed		1.657869523
*p* (*T* ≤ *t*) two-tailed		0.007693381
*t*, critical two-tailed		1.980272226

*t*-test: two independent samples assuming unequal variances.

## Discussion

The results show noteworthy beneficial changes on certain HRV parameters from the Heart+ App compared with the sham app. There is a statistically significant reduction in LF/HF, a trend in LF, and a possible trend in RMSSD, which taken collectively suggest that the Heart+ App has a positive influence on HRV, especially on ANS balance by reducing sympathetic dominance. These results suggest improved physiological regulation of the heart and ANS balance with improved resilience to stress. ANS balance is also an important prerequisite for healing. The results obtained here are consistent with those of previous studies on SRT using analog devices imbued with frequency information, which showed beneficial health effects on a variety of living systems, as well as similar beneficial effects on HRV parameters, described earlier. However, this study differs from most of the previous studies on SRT in that the subjects here were not challenged with any imposed environmental stressors; subjects were in a normal resting state. Although the calculated effect sizes in this study were small, clinical significance was shown in the statistical comparison of the post–pre differences in LF/HF for the sham app and the Heart+ App. The Heart+ App shows promising results to impact ANS balance and health positively.

The magnitude of these effects was compared to other short-term interventions. LF/HF decreased by 30.2% (*p* < 0.001) for the Heart+ App, suggesting significantly improved autonomic tone. By comparison, a study on the effects on cardiac autonomic tone of a 20 min myofascial trigger point massage to the upper torso while seated yielded a 17.1% (*p* = 0.04) decrease in LF/HF.^32^ A study on the effects of short-term practice of yoga, including postures, breathing techniques, and meditation, performed for an hour daily for 1 month by healthy adults, showed a reduction in LF/HF of 13% (*p* < 0.05).^33^ Thus, the impact of the Heart+ App on HRV and autonomic tone appears to be more robust than that from short-term stress-reduction interventions such as massage and yoga, which take time and effort to perform. This suggests possible clinical applications of the Heart+ App that merit further study with a larger, more diverse subject population and over a larger set of exposure parameters and multiple assessments.

This study was exploratory to look for any short-term effects on HRV in healthy adults from exposure to the Heart+ App using 5 min data acquisition periods. The time dependence, including the long-term effects of such exposure to the Heart+ App, is unknown. The effect of the technology on patients with diseases is also unknown. Subsequent research and development on the Heart+ App could potentially optimize its beneficial effects. In order to do this, it would be important to characterize the purported non-Hertzian signals that comprise this technology and to conduct basic science studies to understand its *modus operandi*. Real-time monitoring of HRV might elicit the dynamics of the physiologic response to the Heart+ App and help elucidate its underlying means of action.

The study was rigorously controlled to eliminate potential confounding variables. Nonetheless, because multiple HRV parameters were assessed, it is possible that some of the statistically significant findings or trends may be due to chance.

There is some debate over the meaning and utility of LF and LF/HF that requires further elaboration. Short-term recordings showing changes in these parameters can be misinterpreted as changes in sympathetic activity according to some,^31^ especially in studies that involve changes in the breath. Clearly, the present study did not involve activity impacting the breath, as subjects were seated and reading neutral material. Nonetheless, the relationship between the sympathetic and parasympathetic nervous systems appears to be complex and nonlinear. At the very least, changes in LF and LF/HF can be considered changes in the modulation of autonomic tone.^34^

Replication of these findings by this researcher and other investigators using larger samples in studies of longer duration and with multiple exposures to the Heart+ App would be important to confirm and extend these findings. Additional measures to assess ANS balance, stress levels, and human performance would also be important to test with the Heart+ App in future studies, under normal test conditions as well as when challenged by various stressors. Most significantly, the fact that any effect was measured at all has profound implications for subtle energy effects on human physiology. The results of this double-blind sham-controlled trial strongly support the notion that non-Hertzian electromagnetic fields can modulate ANS parameters. These signals are broadcast from a wireless communication device and may not only affect the user, but others nearby. It is possible that similar technologies could be developed for wireless delivery, for example to improve the quality of sleep or energy and performance. A vast range of biological effects may indeed be possible.

## Conclusions

A significant reduction of 30.2% in mean LF/HF (*p* < 0.001), along with possible trends in mean LF decreasing by 7.3% and mean RMSSD increasing by 7.1%, were observed for healthy adult subjects in a resting state following a 35 min exposure to a passive software application, the Heart+ App, working in the background on an active smart phone untouched by the subjects. Therefore, it appears that specific frequencies broadcast over a short distance can be bioactive and beneficially impact ANS regulation, reducing sympathetic dominance, the “fight or flight” response. Such effects of a passive digital technology may help regulate homeodynamics, increasing one's ability to cope with stress, and improving overall well-being, completely effortlessly, which may enhance wellness and performance.
